# Vaccine breakthrough hypoxemic COVID-19 pneumonia in patients with auto-Abs neutralizing type I IFNs

**DOI:** 10.1126/sciimmunol.abp8966

**Published:** 2022-06-14

**Authors:** Paul Bastard, Sara Vazquez, Jamin Liu, Matthew T. Laurie, Chung Yu Wang, Adrian Gervais, Tom Le Voyer, Lucy Bizien, Colin Zamecnik, Quentin Philippot, Jérémie Rosain, Emilie Catherinot, Andrew Willmore, Anthea M. Mitchell, Rebecca Bair, Pierre Garçon, Heather Kenney, Arnaud Fekkar, Maria Salagianni, Garyphallia Poulakou, Eleni Siouti, Sabina Sahanic, Ivan Tancevski, Günter Weiss, Laurenz Nagl, Jérémy Manry, Sotirija Duvlis, Daniel Arroyo-Sánchez, Estela Paz Artal, Luis Rubio, Cristiano Perani, Michela Bezzi, Alessandra Sottini, Virginia Quaresima, Lucie Roussel, Donald C. Vinh, Luis Felipe Reyes, Margaux Garzaro, Nevin Hatipoglu, David Boutboul, Yacine Tandjaoui-Lambiotte, Alessandro Borghesi, Anna Aliberti, Irene Cassaniti, Fabienne Venet, Guillaume Monneret, Rabih Halwani, Narjes Saheb Sharif-Askari, Jeffrey Danielson, Sonia Burrel, Caroline Morbieu, Yurii Stepanovskyy, Anastasia Bondarenko, Alla Volokha, Oksana Boyarchuk, Alenka Gagro, Mathilde Neuville, Bénédicte Neven, Sevgi Keles, Romain Hernu, Antonin Bal, Antonio Novelli, Giuseppe Novelli, Kahina Saker, Oana Ailioaie, Arnau Antolí, Eric Jeziorski, Gemma Rocamora-Blanch, Carla Teixeira, Clarisse Delaunay, Marine Lhuillier, Paul Le Turnier, Yu Zhang, Matthieu Mahevas, Qiang Pan-Hammarström, Hassan Abolhassani, Thierry Bompoil, Karim Dorgham, COVID HGE consortium, French COVID study group, COMET consortium, Guy Gorochov, Cédric Laouenan, Carlos Rodríguez-Gallego, Lisa F. P. Ng, Laurent Renia, Aurora Pujol, Alexandre Belot, François Raffi, Luis M. Allende, Javier Martinez-Picado, Tayfun Ozcelik, Sevgi Keles, Luisa Imberti, Luigi D. Notarangelo, Jesus Troya, Xavier Solanich, Shen-Ying Zhang, Anne Puel, Michael R Wilson, Sophie Trouillet-Assant, Laurent Abel, Emmanuelle Jouanguy, Chun Jimmie Ye, Aurélie Cobat, Leslie M. Thompson, Evangelos Andreakos, Qian Zhang, Mark S. Anderson, Jean-Laurent Casanova, Joseph L. DeRisi

**Affiliations:** 1. Laboratory of Human Genetics of Infectious Diseases, Necker Branch, INSERM U1163, Necker Hospital for Sick Children, Paris, France.; 2. University of Paris Cité, Imagine Institute, Paris, France.; 3. St. Giles Laboratory of Human Genetics of Infectious Diseases, Rockefeller Branch, The Rockefeller University, New York, NY, USA.; 4. Department of Pediatrics, Necker Hospital for Sick Children, AP-HP, Paris, France.; 5. Medical Scientist Training Program, University of California, San Francisco, San Francisco, CA 94143, USA.; 6. Tetrad Graduate Program, University of California, San Francisco, San Francisco, CA 94143, USA.; 7. Diabetes Center, University of California, San Francisco, San Francisco, CA 94143, USA.; 8. Department of Biochemistry and Biophysics, University of California, San Francisco, San Francisco, CA 94158, USA.; 9. University of California, Berkeley-University of California, San Francisco Graduate Program in Bioengineering, University of California, San Francisco, California, United States.; 10. Chan Zuckerberg Biohub, San Francisco, CA 94158, USA.; 11. Weill Institute for Neurosciences, Department of Neurology, University of California, San Francisco, San Francisco, CA, USA.; 12. Pneumology Department, Foch Hospital, Suresne, France.; 13. Intensive Care Unit, Grand Hôpital de l'Est Francilien Site de Marne-la-Vallée, Jossigny, France.; 14. Laboratory of Clinical Immunology and Microbiology, Division of Intramural Research, NIAID, NIH, Bethesda, MD, USA.; 15. Service de Parasitologie-Mycologie, Groupe Hospitalier Pitié Salpêtrière, Assistance Publique-Hôpitaux de Paris (AP-HP), Paris, France.; 16. Laboratory of Immunobiology, Center for Clinical, Experimental Surgery and Translational Research, Biomedical Research Foundation of the Academy of Athens, Athens, Greece.; 17. 3rd Department of Internal Medicine, National and Kapodistrian University of Athens, Medical School, ‘’Sotiria’’ General Hospital of Chest Diseases, Athens, Greece.; 18. Department of Internal Medicine II, Medical University of Innsbruck, Innsbruck, Austria.; 19. Department of Internal Medicine V, Medical University of Innsbruck, Innsbruck, Austria.; 20. Faculty of Medical Sciences, University “Goce Delchev”, Stip, Republic of North Macedonia.; 21. Institute of public health of Republic of North Macedonia.; 22. Department of Immunology, Instituto de Investigación Sanitaria Hospital 12 de Octubre (imas12) and Department of Immunology, Ophthalmology and ENT, Complutense University School of Medicine, CIBERINFEC, Madrid, Spain.; 23. Emergency Room, ASST Spedali Civili di Brescia, Brescia, Italy.; 24. Covid Unit, ASST Spedali Civili, Brescia, Italy.; 25. CREA Laboratory, Diagnostic Department, ASST Spedali Civili di Brescia, Brescia, Italy.; 26. Department of Medicine, Division of Infectious Diseases, McGill University Health Centre, Montréal, Québec, Canada.; 27. Infectious Disease Susceptibility Program, Research Institute-McGill University Health Centre, Montréal, Québec, Canada.; 28. Department of Microbiology, Universidad de La Sabana, Chía, Colombia.; 29. Department of Critical Care Medicine, Clínica Universidad de La Sabana, Chía, Colombia.; 30. Department of Infectious Diseases, Necker Hospital for Sick Children, AP-HP, Paris, France.; 31. Pediatric Infectious Diseases Unit, Bakirkoy Dr. Sadi Konuk Training and Research Hospital, University of Health Sciences, Istanbul, Turkey.; 32. Department of Immunology, Saint-Louis Hospital, AP-HP, Paris, France.; 33. INSERM UMR 1137 IAME, Paris, France.; 34. INSERM UMR 1272 Hypoxie & Poumon, Bobigny, France.; 35. Pneumology and infectiology department, CH Saint Denis, Saint-Denis, France.; 36. Neonatal Intensive Care Unit, Fondazione IRCCS Policlinico San Matteo, Pavia, Italy.; 37. Anesthesia and Intensive Care, Rianimazione I, Fondazione IRCCS Policlinico San Matteo, Pavia, Italy.; 38. Molecular Virology Unit, Microbiology and Virology Department, Fondazione IRCCS Policlinico San Matteo, Pavia, Italy.; 39. Laboratoire d’Immunologie, Hospices Civils de Lyon, Hôpital Edouard Herriot, Lyon, France.; 40. EA 7426, Pathophysiology of Injury-Induced Immunosuppression, Université Claude Bernard Lyon 1, Hospices Civils de Lyon, Hôpital Edouard Herriot-BioMérieux, Lyon, France.; 41. CIRI, INSERM U1111, CNRS, UMR5308, Ecole Normale Supérieure de Lyon, Université Claude Bernard Lyon 1, Lyon, France.; 42. Sharjah Institute for Medical Research, College of Medicine, University of Sharjah, Sharjah, United Arab Emirates.; 43. Immunology Research Laboratory, College of Medicine, King Saud University.; 44. Sorbonne Université, INSERM U1136, Institut Pierre Louis d'Epidémiologie et de Santé Publique (iPLESP), AP-HP, Hôpital Pitié Salpêtrière, Service de Virologie, Paris, France.; 45. Internal medicine department, Louis Mourier Hospital, AP-HP, Paris, France.; 46. Shupyk National Healthcare University of Ukraine, Kyiv, Ukraine.; 47. Department of Children's Diseases and Pediatric Surgery, I.Horbachevsky Ternopil National Medical University, Ternopil, Ukraine.; 48. Department of Pediatrics, Children's Hospital Zagreb, University of Zagreb School of Medicine, Zagreb, Josip Juraj Strossmayer University of Osijek, Medical Faculty Osijek, Osijek, Croatia.; 49. Intensive care unit, Foch Hospital, Suresne, France.; 50. Department of Pediatrics Hematology Immunology and Rheumatology, Necker Hospital for Sick Children, AP-HP, Paris, France.; 51. Meram Medical Faculty, Necmettin Erbakan University, Meram Medical Faculty, Konya, Turkey.; 52. Service des Urgences, Groupement Hospitalier Nord, Hospices Civils de Lyon, Lyon, France.; 53. Laboratoire de virologie, Institut agent infectieux, Groupement Hospitalier Nord, Hospices Civils de Lyon, Lyon, France.; 54. Laboratory of Medical Genetics, IRCCS Bambino Gesù Children’s Hospital, Rome, Italy.; 55. Department of Biomedicine and Prevention, Tor Vergata University of Rome, Rome, Italy.; 56. Joint Research Unit, Hospices Civils de Lyon-bio Mérieux, Hospices Civils de Lyon, Lyon Sud Hospital, Pierre-Bénite, France; International Center of Research in Infectiology, Lyon University, INSERM U1111, CNRS UMR 5308, ENS, UCBL, Lyon, France.; 57. Service de Génétique, Hôpital Raymond Poincaré, AP-HP, Garches, France.; 58. Department of Internal Medicine, Hospital Universitari de Bellvitge, IDIBELL, Barcelona, Spain.; 59. General pediatric department, PCCEI, CeRéMAIA, Univ Montpellier, CHU Montpellier, Montpellier, France.; 60. Unidade de Infeciologia e Imunodeficiências, Centro Materno-infantil do Norte, Centro Hospitalar Universitário do Porto, Porto, Portugal.; 61. Department of Infectious Diseases, CHU Nantes, and INSERM UIC 1413, CHU Nantes, France.; 62. Geriatric department, CHU Nantes, Hopital Bellier, Nantes, France.; 63. NIAID Clinical Genomics Program, National Institutes of Health, Bethesda, USA; 64. Necker Enfants Malades Institute (INEM), INSERM U1151/CNRS UMR 8253, University of Paris Cité, Paris, France.; 65. Departement of Internal Medicine, Henri Mondor University Hospital, Assistance Publique-Hôpitaux de Paris (AP-HP), Paris-Est Créteil University (UPEC), Créteil, France.; 66. INSERM U955, team 2. Mondor Biomedical Research Institute (IMRB), Paris-Est Créteil University (UPEC), Créteil, France.; 67. Department of Biosciences and Nutrition, Karolinska Institutet, SE14183, Huddinge, Sweden.; 68. Biologie/Pathologie, CHU-Nantes - Hôtel Dieu, Institut de Biologie, Nantes, France; 69. Sorbonne Université, Inserm, Centre d’Immunologie et des Maladies Infectieuses, (CIMI- Paris), Paris, France.; 70. Département d’Immunologie, Assistance Publique Hôpitaux de Paris (AP-HP), Hôpital Pitié-Salpétrière, Paris, France.; 71. Université de Paris, IAME UMR-S 1137, INSERM, Paris, France.; 72. AP-HP, Département Epidémiologie Biostatistiques et Recherche Clinique, Hôpital Bichat, Paris, France.; 73. Department of Clinical Sciences, University Fernando Pessoa Canarias, Las Palmas de Gran Canaria, Canary Islands, Spain.; 74. Department of Immunology, University Hospital of Gran Canaria Dr. Negrín, Canarian Health System, Las Palmas de Gran Canaria, Spain.; 75. A*STAR Infectious Disease Labs, Agency for Science, Technology and Research, Singapore.; 76. Lee Kong Chian School of Medicine, Nanyang Technology University, Singapore.; 77. School of Biological Sciences, Nanyang Technology University, Singapore.; 78. Neurometabolic Diseases Laboratory, IDIBELL-Hospital Duran i Reynals, CIBERER U759, and Catalan Institution of Research and Advanced Studies (ICREA), Barcelona, Spain.; 79. CNRS UMR 5308, ENS, UCBL, Lyon, France; National Referee Centre for Rheumatic, and Autoimmune and Systemic Diseases in Children (RAISE), Lyon, France; Lyon; Immunopathology Federation LIFE, Hospices Civils de Lyon, Lyon, France.; 80. IrsiCaixa AIDS Research Institute and Institute for Health Science Research Germans Trias i Pujol (IGTP), Badalona, Spain.; 81. Infectious Diseases and Immunity, Center for Health and Social Care Research (CESS), Faculty of Medicine, University of Vic-Central University of Catalonia (UVic-UCC), Vic, Spain.; 82. Catalan Institution for Research and Advanced Studies (ICREA), Barcelona, Spain.; 83. CIBER de Enfermedades Infecciosas, Instituto de Salud Carlos III, Madrid, Spain.; 84. Department of Molecular Biology and Genetics, Bilkent University, Bilkent - Ankara, Turkey.; 85. Department of Internal Medicine, Infanta Leonor University Hospital, Madrid, Spain.; 86. Hospices Civils de Lyon, Lyon, France; International Center of Research in Infectiology, Lyon University, INSERM U1111, CNRS UMR 5308, ENS, UCBL, Lyon, France.; 87. ImmunoX Initiative, University of California, San Francisco, San Francisco, CA 94143, USA.; 88. Departments of Epidemiology and Biostatistics and Bioengineering and Therapeutic Sciences, University of California, San Francisco, San Francisco, CA 94143, USA.; 89. Bakar Computational Health Sciences Institute, University of California, San Francisco, San Francisco, CA 94143, USA.; 90. Parker Institute for Cancer Immunotherapy, San Francisco, CA 94129, USA.; 91. Departments of Psychiatry and Human Behavior and Neurobiology and Behavior, University of California, Irvine, CA, USA.; 92. Division of Endocrinology and Metabolism, Department of Medicine, University of California, San Francisco, San Francisco, CA 94143, USA.; 93. Howard Hughes Medical Institute, New York, NY, USA.

## Abstract

Life-threatening ‘breakthrough’ cases of critical COVID-19 are attributed to poor or waning antibody response to the SARS-CoV-2 vaccine in individuals already at risk. Pre-existing autoantibodies (auto-Abs) neutralizing type I IFNs underlie at least 15% of critical COVID-19 pneumonia cases in unvaccinated individuals; however, their contribution to hypoxemic breakthrough cases in vaccinated people remains unknown. Here, we studied a cohort of 48 individuals (age 20-86 years) who received 2 doses of an mRNA vaccine and developed a breakthrough infection with hypoxemic COVID-19 pneumonia 2 weeks to 4 months later. Antibody levels to the vaccine, neutralization of the virus, and auto-Abs to type I IFNs were measured in the plasma. Forty-two individuals had no known deficiency of B cell immunity and a normal antibody response to the vaccine. Among them, ten (24%) had auto-Abs neutralizing type I IFNs (aged 43-86 years). Eight of these ten patients had auto-Abs neutralizing both IFN-α2 and IFN-ω, while two neutralized IFN-ω only. No patient neutralized IFN-β. Seven neutralized 10 ng/mL of type I IFNs, and three 100 pg/mL only. Seven patients neutralized SARS-CoV-2 D614G and the Delta variant (B.1.617.2) efficiently, while one patient neutralized Delta slightly less efficiently. Two of the three patients neutralizing only 100 pg/mL of type I IFNs neutralized both D61G and Delta less efficiently. Despite two mRNA vaccine inoculations and the presence of circulating antibodies capable of neutralizing SARS-CoV-2, auto-Abs neutralizing type I IFNs may underlie a significant proportion of hypoxemic COVID-19 pneumonia cases, highlighting the importance of this particularly vulnerable population.

## Teaser

Type I IFN auto-Abs are found in 20% of hypoxemic, mRNA vaccinated COVID-19 patients despite SARS-CoV-2 neutralizing antibodies.

## INTRODUCTION

Since the start of the coronavirus disease 19 (COVID-19) pandemic (*1*), caused by severe respiratory syndrome coronavirus 2 (SARS-CoV-2), at least 6 million people have died from COVID-19 (*2*). Although the majority of infected individuals recover, it remains important to identify factors that put patients at greater risk for severe disease. Age is the major epidemiological risk factor of death from pneumonia, the risk doubling every five years of age from childhood onward (*3*–*5*). Patients with inborn errors (IE) of immunity affecting the production of, or response to type I IFNs, or both, are prone to critical COVID-19 pneumonia (*6*–*8*). These findings established the crucial role of type I IFNs in fending off SARS-CoV-2 (*9*). Moreover, auto-Abs neutralizing high concentrations (10 ng/mL in plasma diluted 1/10) of IFN-α2 and/or IFN-ω were found in at least 10% of individuals with critical COVID-19 (*10*), an observation replicated in various regions of the world (*11*–*21*). Patients with autoimmune polyendocrine syndrome type I (APS-1) harbor these neutralizing auto-Abs from early childhood and are at high risk of life-threatening COVID-19 (*20*, *21*). Moreover, at least 13.6% of unvaccinated patients with critical COVID-19 had auto-Abs neutralizing lower, more physiological concentrations (100 pg/mL in plasma diluted 1/10) of IFN-α2 and/or IFN-ω, while auto-Abs neutralizing IFN-β were found in another 1% of patients (*22*). In more than 34,000 uninfected individuals aged 18 to 100 years, the prevalence of auto-Abs neutralizing 10 ng/mL (or 100 pg/mL) of IFN-α2 or IFN-ω increased significantly with age, with 0.17% (1.1%) of individuals positive for these auto-Abs under 70 years old, and more than 1.4% (4.4%) positive over 70 years old, consistent with the higher risk of life-threatening COVID-19 in the elderly population (*22*). These auto-Abs thus precede infection and are strong determinants of critical disease, only second to age among common risk factors (*23*). The odds ratios (ORs) of critical disease are the highest in individuals with auto-Abs neutralizing 10 ng/mL of both IFN-α2 and IFN-ω (OR = 67; p-value = 7.8x10^−13^) (*22*, *23*).

RNA vaccines are highly effective at protecting against severe COVID-19 pneumonia (*24*, *25*). Despite their efficacy, ‘breakthrough’ cases, i.e., individuals diagnosed with SARS-CoV-2 infection despite being vaccinated with 2 doses, have been reported worldwide (*26*, *27*). Most breakthrough cases are asymptomatic or mild (*26*), but in rare cases they are severe, critical, or even fatal (*28*, *29*). It is thought that these severe or critical cases can result from a pathologically deficient (including inherited and acquired deficiencies of adaptive immunity) or a physiologically waning antibody response to the vaccine (especially in aging individuals). Incomplete protection from viral genotypes with vaccine-resilient mutations (such as Delta or Omicron), can also result in insufficient viral neutralization in vivo, in individuals otherwise at risk of hypoxemic pneumonia (for example, due to their age, sex, co-morbidity, rare or common genetic variant, or auto-Abs to type I IFNs) (*30*). In other words, breakthrough critical cases are thought to be due to a poor antibody response to the vaccine in at-risk individuals (*31*). Yet, the human genetic and immunological determinants of critical ‘breakthrough’ cases remain unclear, especially in patients with normal antibody response to the vaccine. Moreover, the biological and clinical efficacy of RNA vaccines in patients with known genetic or immunological determinants of critical COVID-19 pneumonia, i.e., in patients with IE of, or auto-Abs to type I IFNs, is not clear. With the COVID Human Genetic Effort (CHGE, www.covidhge.com), we recruited and tested patients with breakthrough COVID-19 and hypoxemic pneumonia. We tested the double hypothesis that some of these breakthrough cases of severe or critical COVID-19 pneumonia may have a normal antibody response to the vaccine and may also harbor auto-Abs to type I IFNs.

## RESULTS

### Fourty-two of 48 patients have normal antibody response to the vaccine

**Fig. 1.**
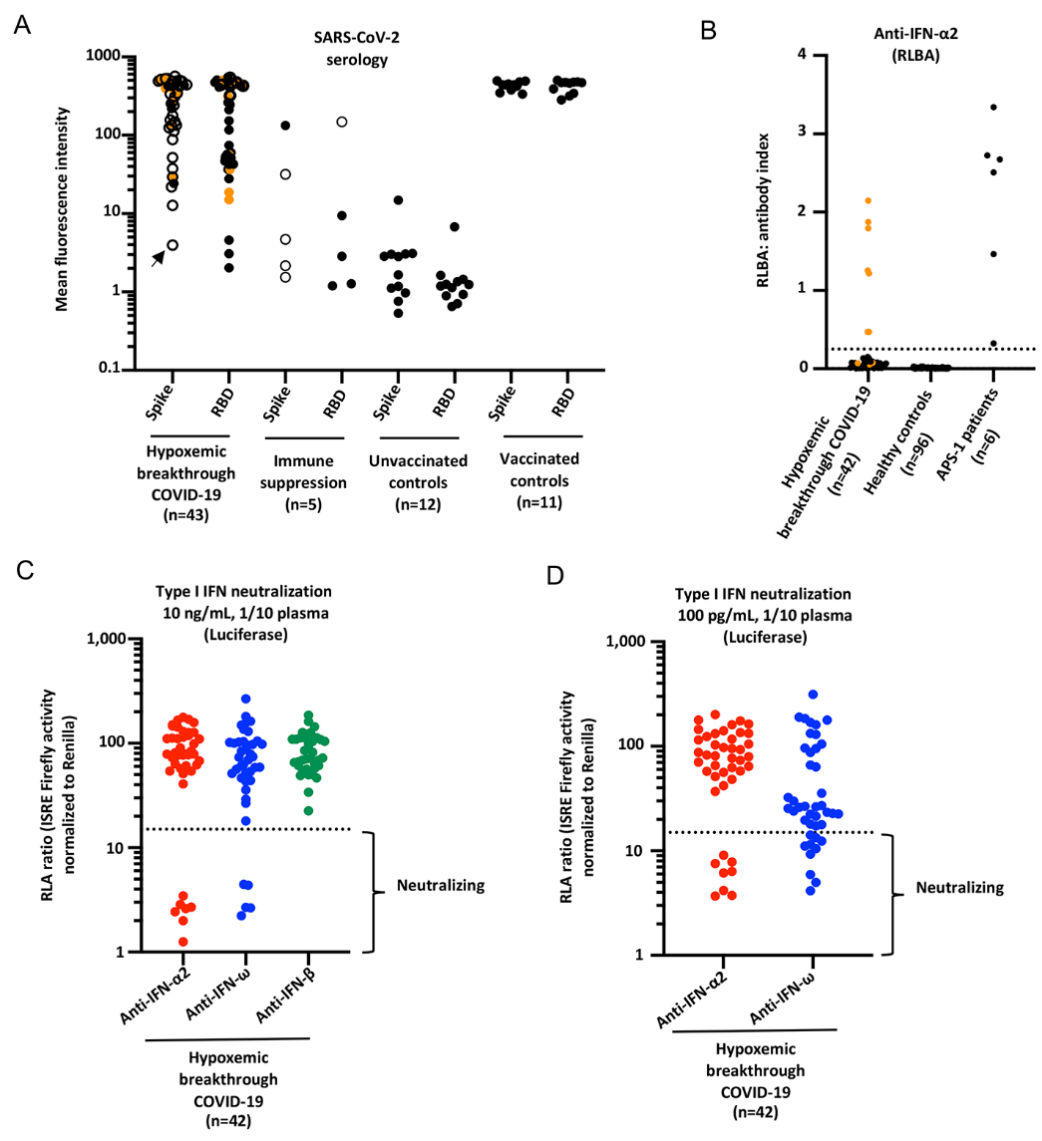
**Neutralizing auto-antibodies (Abs) against IFN-α2 and IFN-ω in patients with hypoxemic breakthrough COVID-19 despite a normal serological response to SARS-CoV-2 mRNA vaccine. (A)** SARS-CoV-2 serology against spike(S)-protein and receptor binding domain (RBD) in hypoxemic breakthrough COVID-19 (N=43), patients with immune suppression (n=5), unvaccinated controls (N=12), and vaccinated and uninfected healthy controls (n=11). Mean fluorescence intensity is shown. The orange dots correspond to the 10 individuals with auto-Abs neutralizing type I IFNs. Empty circles represent either Spike or RBD serology, to outline the highest value for one patient. The arrow represents the patient without B cell deficiency but with an insufficient Ab response to the virus. **(B)** Radioligand binding assay (RLBA) results for auto-Abs against IFN-α2 in patients with hypoxemic breakthrough COVID-19 pneumonia without immune suppression or low Ab response to the vaccine (N=42), uninfected controls (N=96), and uninfected APS-1 patients (N=6). **(C)** Neutralization of 10 ng/mL IFN-α2, IFN-ω or IFN-β in the presence of plasma 1/10 from patients with hypoxemic breakthrough COVID-19 pneumonia with a good Ab response to the vaccine (N=42). Relative luciferase activity is shown (ISRE dual luciferase activity, with normalization against *Renilla* luciferase activity) after stimulation with 10 ng/mL IFN-α2 or IFN-ω in the presence of plasma 1/10. RLA: relative luciferase activity. **(D)** Neutralization of 100 pg/mL IFN-α2 or IFN-ω in the presence of plasma 1/10 from patients with hypoxemic breakthrough COVID-19 pneumonia with a good Ab response to the vaccine (N=42).

Forty-eight patients who suffered from hypoxemic COVID-19 pneumonia (severe or critical), despite having received 2 doses of mRNA vaccine, at least 2 weeks and up to 16 weeks (mean: 8 weeks) before infection were recruited from 6 countries (France, Greece, North Macedonia, Turkey, Ukraine, and United States of America). All COVID Human Genetic Effort (CHGE) patients whose samples were available were recruited; they had not been previously infected with SARS-CoV-2, as attested by the clinical information collected and/or a negative serology at the time of vaccination or performed at the onset of disease. These patients were aged 20 to 86 years (mean 53 years old) and included 34 men and 14 women. Five of them had a known deficiency of B cell immunity (immunosuppressive therapy in 3 individuals, and HIV infection in 1, and lymphoma with CAR-T cell treatment in one). We tested the 48 patients for their antibody response to SARS-CoV-2 mRNA vaccines. We found one of the 43 patients did not have a known B cell deficiency, but had an insufficient antibody response to the vaccine (defined as within 3 standard deviations from the mean of unvaccinated controls) (Arrow, Fig. 1A, S1A). The other patients had levels of antibody response to the vaccine similar to those of vaccinated controls (t-test, Supplementary Table 1). Of note, 3 of the 5 patients with a known B cell deficiency had a normal antibody response (above 3 standard deviations) (Fig. 1A). Overall, 42 patients had both no B cell deficiency and a normal antibody response to the vaccine, thus were further investigated.

### Auto-Abs against type I IFNs in 10 of 42 patients with normal Ab response to the vaccine

We next tested all the samples from the 42 patients without known B cell deficiency and with a normal Ab response to the mRNA vaccine for IgG auto-Ab to type I IFN levels using a radioligand binding assay (RLBA). Seven of 42 patients tested had elevated titers of anti-IFN-α2 auto-Abs in RLBA (Fig. 1B). We then tested all these samples for their neutralization activity against IFN-α2, IFN-, and IFN-β at 10 ng/mL, 100 pg/mL, and 10 ng/mL respectively. We identified ten (24%) patients with IgG auto-Abs neutralizing IFN-α2 and/or IFN-ω, as did the APS-1 positive controls, while the healthy controls did not (Fig. 1C, D). Patients with neutralizing auto-Abs have lower luciferase induction (below threshold in dotted lines). All these patients had normal anti-SARS-CoV-2 Spike antibody response to the vaccine (Fig. S1D, E). In contrast, auto-Abs to type I IFN were not found in any of the 6 patients previously excluded because of a known B cell immunodeficiency (n=5) or an insufficient antibody response to the vaccine (n=1) (Fig. S1B, C). Of note, 8 of these 10 individuals (80%) had circulating auto-Abs neutralizing both IFN-α2 and IFN-ω, while two neutralized IFN-ω only (20%), and none neutralized IFN-β (Fig. 1C-D and Table 2). In addition, plasma from 7 patients (diluted 1/10) neutralized a high concentration (10 ng/mL) of type I IFNs (70%), while 3 neutralized only the lower, more physiological, dose (100 pg/mL) of type I IFNs (including the 2 neutralizing IFN-ω only) (30%) (Fig. 1C, D and Table 2). Overall, auto-Abs neutralizing IFN-α2 and/or IFN-ω were found at the onset of disease in 10 of 42 patients (24%) with breakthrough COVID-19 who suffered from hypoxemic pneumonia, despite having a normal antibody response to an mRNA vaccine.

**Table 2.** Auto-Abs neutralized in the 10 patients. 1: neutralizing. 0: non-neutralizing.

**Patient**	**anti-IFN-α2 auto-Abs (10 ng/mL)**	**anti-IFN-β auto-Abs (10 ng/mL)**	**anti-IFN-ω, auto-Abs (10 ng/mL)**	**anti-IFN-α2 auto-Abs (100 pg/mL)**	**anti-IFN-ω, auto-Abs (100 pg/mL)**
**P1**	1	0	1	1	1
**P2**	1	0	0	1	1
**P3**	1	0	0	1	1
**P4**	0	0	0	0	1
**P5**	1	0	1	1	1
**P6**	0	0	0	1	1
**P7**	0	0	0	0	1
**P8**	1	0	1	1	1
**P9**	1	0	1	1	1
**P10**	1	0	1	1	1

### Demographic, clinical, and virological features of the 10 patients with auto-Abs to type I IFNs

The patients with hypoxemic breakthrough COVID-19 pneumonia and auto-Abs neutralizing type I IFNs included three women and seven men. They were aged 43 to 86 years old (mean: 75 years old) (Table 1). All were of European ancestry, except one Cambodian, and they originated from France (n=3), Greece (n=5), and the USA (n=2). None of these individuals reported having previously suffered from other severe viral infections. All 10 patients were hospitalized during COVID-19 for oxygen supplementation, including 5 hospitalized in an intensive care unit (ICU) who received mechanical ventilation, and one who received nasal oxygen high flow therapy but was recused of ICU because of age (P8). All of them survived. All presented with bilateral COVID-19 pneumonia and had a positive SARS-CoV-2 RT-PCR in the respiratory tract. The SARS-CoV-2 variants involved were unknown but most likely to be Delta variant, given the epidemiology at the location and time of sampling (i.e., before October 2021 for all samples tested). They had been vaccinated 2 to 16 weeks prior to the diagnosis of COVID-19. Of note, one individual (P2) had at least two auto-immune conditions (myasthenia gravis and Hashimoto’s thyroiditis), while another (P10) had APS-1. Myasthenia gravis and APS-1 are associated with auto-Abs to type I IFNs, which had however not been measured prior to COVID-19 in these two individuals. Finally, one individual (P1) belonged to a large family, whose members had all been fully vaccinated, and many were infected at the same time as he did (*32*). He was nevertheless the only one to suffer from critical disease, and also the only one to harbor neutralizing auto-Abs to type I IFNs. None of the 10 patients died of COVID-19, while more than 20% of unvaccinated individuals who died of COVID-19 harbored neutralizing auto-Abs (*22*) and 5-10% of unvaccinated patients with these auto-Abs died of COVID-19 (*23*), suggesting that although insufficient to prevent hypoxemic pneumonia, vaccination may have protected these patients from a fatal outcome. Overall, auto-Abs to type I IFNs can underlie hypoxemic breakthrough COVID-19 infection in previously healthy individuals who developed normal antibody responses after SARS-CoV-2 mRNA vaccination.

**Table 1.** Clinical and demographic information of the 10 patients with hypoxemic breakthrough COVID-19 infection and auto-Abs neutralizing type I IFNs. HTN: hypertension, AF: atrial fibrillation. APS-1: auto-immune polyendocrine syndrome type 1.

**Patient**	**Origin**	**Residence**	**Sex**	**Age**	**Comorbidities**	**Vaccine source**	**Doses number**	**Time of disease post vaccination (weeks)**	**ICU**	**Classification**	**Outcome**
**P1**	American	USA	M	80	Diabetes, asthma	Pfizer	2	2	Yes	Critical	Alive
**P2**	Greek	Greece	F	82	HTN, myasthenia gravis, hashimoto, dyslipidemia	Pfizer	2	4	Yes	Critical	Alive
**P3**	Greek	Greece	M	73	HTN, diabetes, dyslipidemia, glaucome	Pfizer	2	2	Yes	Critical	Alive
**P4**	Greek	Greece	M	86	HTN, diabetes, dyslipidemai, AF, benign prostate hyperplasia, parkinson	Pfizer	2	12	Yes	Critical	Alive
**P5**	Greek	Greece	M	73	Diabetes, coronary heart disease	Pfizer	2	3	No	Severe	Alive
**P6**	Greek	Greece	F	77	HTN, diabetes, dyslipidemia	Pfizer	2	16	No	Severe	Alive
**P7**	Cambodian	France	M	71	HTN	Pfizer	2	15	Yes	Critical	Alive
**P8**	French	France	F	86	NA	Pfizer	2	6	No	Critical	Alive
**P9**	American	USA	M	80	NA	Pfizer	2	2	No	Critical	Alive
**P10**	French	France	M	43	APS-1	Pfizer	2	2	No	Severe	Alive

### Antibodies neutralizing SARS-CoV-2 in all 10 patients

**Fig. 2.**
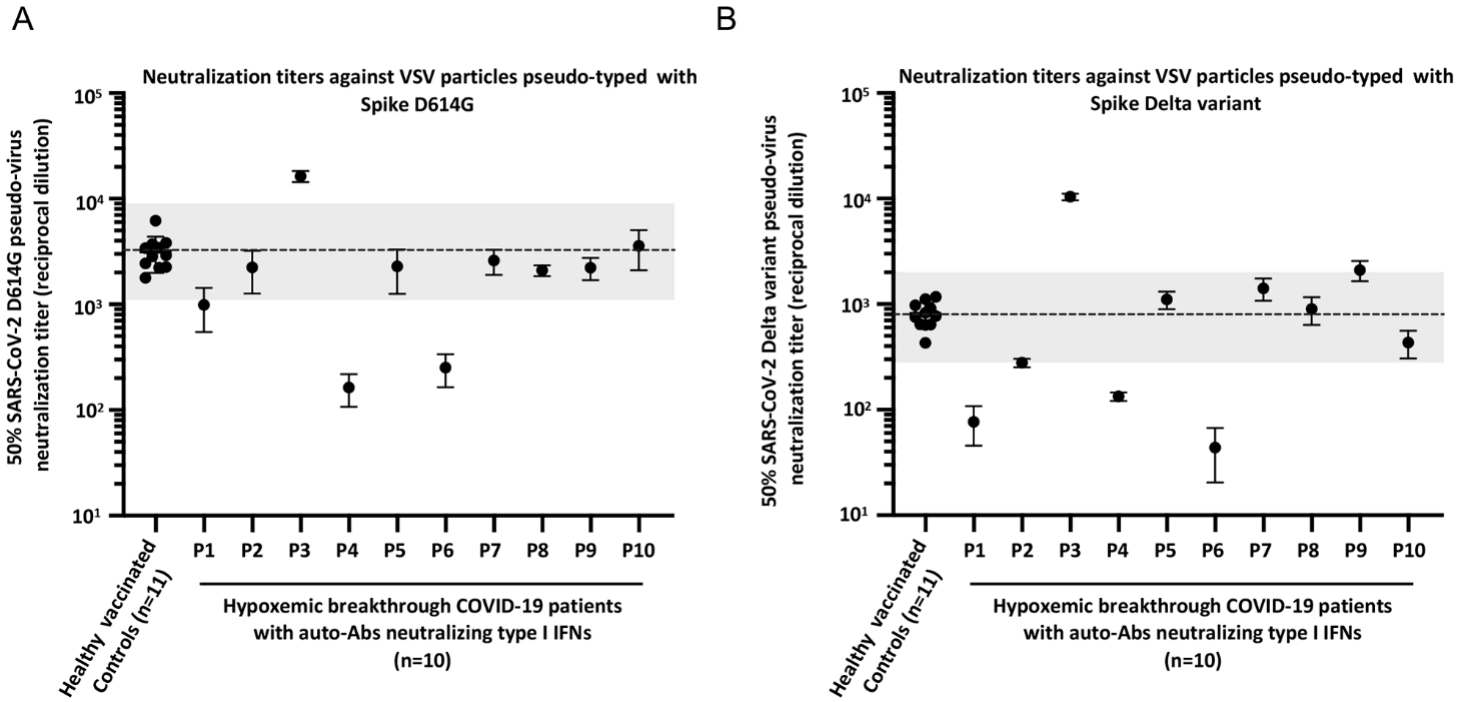
**Neutralization titers against SARS-CoV-2 in the patients with auto-Abs against type I IFNs**. Neutralization titers against SARS-CoV-2 for healthy vaccinated donors 2-8 weeks after the second dose of mRNA vaccine (n=11), and patients with hypoxemic breakthrough COVID-19 pneumonia and auto-Abs to type I IFNs (n=10). The dashed line shows the geometric mean of healthy donor titers, the box shows interquartile range, and the shaded region is the full range. **(A)** Neutralization assay performed with pseudoviruses representing the D614G strain, and **(B)** the Delta variant (B.1.617.2).

To further test the hypothesis that the hypoxemic breakthrough cases were driven by the auto-Abs neutralizing type I IFNs and not by an insufficient antibody response to the vaccine, we assessed the neutralizing activity in all 10 patients’ plasma against SARS-CoV-2. Although we did not collect blood samples prior to COVID-19 diagnosis, we collected them in the first 3 days of hospitalization. As we did not determine with which viral strain the patients had been infected, we performed the neutralization assay with pseudoviruses representing both the previously globally dominant D614G strain and the Delta variant (B.1.617.2), which was dominant when and where the patients were infected. We compared the patients’ results with the neutralization titers of healthy vaccinated donors 2-8 weeks after the 2^nd^ dose of the mRNA vaccine. All 10 individuals tested had a neutralization capacity, when compared with the healthy vaccinated controls, although it was slightly reduced for 2 individuals (P4 and P6) for the D614G strain and for 3 individuals (P1, P4 and P6) for the Delta variant (Fig. 2A, B, S1D, E). Although P1 neutralized 10 ng/mL of type I IFNs, P4 and P6 only neutralized low concentrations of type I IFNs. Specifically, P4 neutralized both IFN-α2 and IFN-ω but only at 100 pg/mL, while P6 neutralized only IFN-ω at 100 pg/mL. This observation suggests that in patients whose auto-Abs neutralized only low concentrations of type I IFNs, sub-optimal antibody response to the vaccine may have also contributed to hypoxemic pneumonia. Overall, this suggested that hypoxemic COVID-19 pneumonia can occur in individuals with a normal antibody reponse to two doses of mRNA vaccine (42 of 48 patients tested). Moreover, in about 20% of the beakthrough cases (10 of 42 cases), hypoxemic pneumonia was probably due to auto-Abs neutralizing IFN-α2 and/or IFN-ω (and typically at high concentration of both IFNs). Finally, 70% of the latter cases (7 of 10 cases), plasma neutralization of two viral strains was normal, while one had a lower neutralization against the delta strain, and the remaining 2 had a subnormal neutralization of both viral strains (D614G, and Delta).

## DISCUSSION

The pathogenesis of life-threatening COVID-19 pneumonia involves two steps, with a deficiency of respiratory type I IFN immunity in the first days of infection resulting in viral spread, which triggers excessive systemic and pulmonary inflammation (*30*, *33*, *34*). The vaccination of billions of individuals has efficiently reduced the number of critical cases. Nevertheless, breakthrough hypoxemic COVID-19 pneumonia can occur in previously healthy individuals who are vaccinated against SARS-CoV-2, which is assumed to be due to a poor antibody response to the vaccine (*31*). Our findings suggest that most breakthrough hypoxemic cases (42 of 48 tested) did not have a known B cell deficiency and also had a normal antibody response to the vaccine, although no samples were available before SARS-CoV-2 infection. Moreover, we showed that about 20% (10 of 42) of these breakthrough cases with normal antibody response to the vaccine also carried auto-Abs neutralizing IFN-α2 and/or IFN-ω (10 ng/mL for 7 patients and 100 pg/mL for 3 patients). In addition, the plasma of 7 of the 10 patients with auto-Abs to type I IFNs efficiently neutralized SARS-CoV-2 in vitro*,* while one had a lower neutralization against the delta strain, and plasma from the remaining 2 neutralized the two viral strains tested sub-optimally. Both patients had auto-Abs neutralizing only 100 pg/mL of type I IFNs. Plasma (diluted 1/10) from seven of the 10 individuals with these auto-Abs neutralized a high concentration (10 ng/mL) of both IFN-α2 and IFN-ω, consistent with unvaccinated individuals carrying such auto-Abs being at the greatest risk of critical COVID-19 among individuals carrying any combinations of auto-Abs to type I IFNs (*22*, *23*, *30*). The proportion of individuals with hypoxemic COVID-19 due to neutralizing both IFN-α2 and IFN-ω at the high dose (10 ng/mL) is even higher in the breakthrough cohort reported here (7 of 42, 16%) than in the previously described unvaccinated cohort (175 of 3,136, 7.1%) (*P =* 0.015) (*22*). Two of the 3 patients neutralizing only 100 pg/mL of type I IFNs, also had a slightly diminished neutralization capacity against SARS-CoV-2, suggesting in these individuals a combination of 2 factors: the presence of auto-Abs to low concentration of type I IFNs, and a suboptimal antibody response to the vaccine.

Nevertheless, as we were not able to identify and study auto-Ab positive individuals who were vaccinated and efficiently protected against severe infection, we cannot estimate the percentage of breakthrough cases with hypoxemic pneumonia in individuals with auto-Abs neutralizing type I IFNs infected with SARS-CoV-2. Until 70 years old, the proportion of individuals from the general population sampled prior to the pandemic that carry auto-Abs against both IFN-α2 and IFN-ω is 0.02% and 0.03% for the neutralization of 10 ng/mL and 100 pg/mL, respectively, while it reaches 0.6% and 1.6% over 70 years old. As mRNA vaccines have high efficacy to prevent critical pneumonia, it is probable that most patients with auto-Abs against type I IFNs benefit from vaccination, although the protection might not be sufficient in individuals neutralizing high concentrations of multiple type I IFNs. It is also not unreasonable to speculate that, despite an infection with a vaccine-covered viral variant and a normal antibody response to the vaccine, a small proportion of the patients with such auto-Abs might not be fully protected by the vaccine, especially if infected with a high viral inoculum. By inference from previous studies, the auto-Abs of the 8 patients neutralizing IFN-α2 also probably neutralizes the 13 types of IFN-α (*10*, *20*, *22*, *35*, *36*). These findings suggest that a potent post-vaccine humoral immunity can be insufficient to fight SARS-CoV-2 infection, especially in patients with auto-Abs neutralizing both IFN-α2 and IFN-ω, and even more so at high concentration.

Our results here suggest it may be beneficial to test for auto-Abs to type I IFN in vaccinated patients diagnosed with breakthrough COVID-19 pneumonia of varying severity. Testing uninfected people, including vaccinated individuals, may also be considered, especially in those over 70 years old given the high prevalence of auto-Abs to type I IFNs in this population (>4%) and their lower global type I IFN immunity (*30*, *36*). One of the 10 patients suffered from APS-1 and thus most likely harbored these auto-Abs since early childhood (*20*, *21*, *37*), while another patient had myasthenia gravis, which is also commonly associated with these auto-Abs (*38*). Testing patients with conditions known to be associated with these auto-Abs may benefit these patients. All individuals with auto-Abs to IFNs might benefit not only from vaccine boosters but perhaps from recurrent vaccinations. Prospective studies assessing vaccine-induced immunity before infection in patients with auto-Abs to type I IFNs would be informative, for example in the setting of vaccine trials. Systematic screening at hospital admission for auto-Abs to type I IFNs would also be of help for the management of vaccinated or unvaccinated individuals with hypoxemic pneumonia. Indeed, monoclonal antibodies (mAbs) neutralizing the virus could also be administered promptly (*39*), as shown for an IRF9-deficient patient (*40*), especially in patients with the highest titers of auto-Abs to type I IFNs. Anti-viral compounds, such as remdesivir (*41*, *42*) or molnupiravir (*43*), may also benefit these patients if administered early in the course of infection. Conversely, in ambulatory patients with these auto-Abs, early recombinant IFN-β therapy may also be considered, to prevent the development of hypoxemic pneumonia (*44*). In sum, our findings indicate that auto-Abs to type I IFNs is a susceptibility factor for a severe clinical course of COVID-19 even in vaccinated subjects with a breakthrough infection.

## MATERIALS AND METHODS

### Study Design

We enrolled 48 patients with proven hypoxemic COVID-19 pneumonia, 12 unvaccinated controls, and 11 vaccinated controls from 6 countries in this study. We collected plasma or serum samples for all these individuals to test for the presence of IgG Abs against SARS-CoV-2 and auto-Abs to type I IFNs by immuno-assay. All individuals were recruited according to protocols approved by local Institutional Review Boards (IRBs).

### COVID-19 classification

The severity of COVID-19 was assessed for each patient as follows (*6*, *10*): “critical COVID-19 pneumonia” was defined as pneumonia developing in patients with critical disease, whether pulmonary, with high-flow oxygen, mechanical ventilation (continuous positive airway pressure, bilevel positive airway pressure, intubation), septic shock, or with damage to any other organ requiring admission to the intensive care unit. “Severe COVID-19” was defined as pneumonia developing in patients requiring low-flow oxygen (<6L/min). The controls were individuals infected with SARS-CoV-2 (as demonstrated by a positive PCR and/or serological test and/or displaying typical symptoms, such as anosmia/ageusia after exposure to a confirmed COVID-19 case) who remained asymptomatic or developed mild, self-healing, ambulatory disease with no evidence of pneumonia.

### Statistics

For comparison of groups in Fig. 1a, a two-sided *t* test was performed using a Python library (SciPy) for both Spike and RBD. Briefly, all groups were compared to the unvaccinated control group (n=12). In addition, the group of auto-Ab positive breakthrough cases were compared to the group of auto-Ab negative breakthrough cases.

### Detection of anti-cytokine auto-Abs by a high throughput automated ELISA (Gyros)

Cytokines, recombinant human (rh)IFN-α2 (Milteny Biotec, ref. number 130-108-984) or rhIFN-ω (Merck, ref. number SRP3061), were first biotinylated with EZ-Link Sulfo-NHS-LC-Biotin (Thermo Fisher Scientific, cat. number A39257), according to the manufacturer’s instructions, with a biotin-to-protein molar ratio of 1:12. The detection reagent contained a secondary antibody Alexa Fluor 647 goat anti-human IgG (Thermo Fisher Scientific, ref. number A21445) diluted in Rexip F (Gyros Protein Technologies, ref. number P0004825; 1/500 dilution of the 2 mg/mL stock to yield a final concentration of 4 μg/mL). Buffer PBS-T 0.01% and Gyros Wash buffer (Gyros Protein Technologies, ref. number P0020087) were prepared according to the manufacturer’s instructions. Plasma or serum samples were then diluted 1/100 in PBS-T 0.01% and tested with the Bioaffy 1000 CD (Gyros Protein Technologies, ref. number P0004253), and the Gyrolab X-Pand (Gyros Protein Technologies, ref. number P0020520). Cleaning cycles were performed in 20% ethanol.

### RLBA for anti-IFN-α2 auto-Ab detection

A DNA plasmid containing full-length cDNA sequence with a Flag-Myc tag (OriGene, #RC221091) was verified by Sanger sequencing and used as template in T7-promoter–based in vitro transcription/translation reactions (Promega, #L1170) using [S35]-methionine (PerkinElmer, #NEG709A). IFN-α2 protein was column-purified using NAP-5 columns (GE Healthcare, #17-0853-01); incubated with 2.5 μl of serum, 2.5 μl of plasma, or 1 μl of anti-myc–positive control antibody (Cell Signaling Technology, #2272); and immunoprecipitated with Sephadex protein A/G beads (4:1 ratio; Sigma-Aldrich, #GE17-5280-02 and #GE17-0618-05) in 96-well polyvinylidene difluoride filtration plates (Corning, #EK-680860). The radioactive counts [counts per minute (cpm)] of immunoprecipitated protein were quantified using a 96-well MicroBeta TriLux liquid scintillation plate reader (PerkinElmer). Antibody index for each sample was calculated as follows: (sample cpm value – mean blank cpm value)/(positive control antibody cpm value – mean blank cpm value). For the COVID-19 patient and CCP cohorts, a positive signal was defined as greater than 6 standards deviations above the mean of pre–COVID-19 blood bank non-inflammatory controls.

### Functional evaluation of anti-cytokine auto-Abs by luciferase reporter assays

The blocking activity of anti-IFN-α2 and anti-IFN-ω auto-Abs was determined with a reporter luciferase activity. Briefly, HEK293T cells were transfected with a plasmid containing the *Firefly* luciferase gene under the control of the human *ISRE* promoter in the pGL4.45 backbone, and a plasmid constitutively expressing *Renilla* luciferase for normalization (pRL-SV40). Cells were transfected in the presence of the X-tremeGene9 transfection reagent (Sigma-Aldrich, ref. number 6365779001) for 24 hours. Cells in Dulbecco’s modified Eagle medium (DMEM, Thermo Fisher Scientific) supplemented with 2% fetal calf serum (FCS) and 10% healthy control or patient serum/plasma (after inactivation at 56°C, for 20 min) were either left unstimulated or were stimulated with IFN-α2 (Milteny Biotech, ref. number 130-108-984), IFN-ω (Merck, ref. number SRP3061), at 10 ng/mL or 100 pg/mL, or IFN-β (Milteny Biotech, ref. number: 130-107-888) at 10 ng/mL, for 16 hours at 37°C. Each sample was tested once for each cytokine and dose. Finally, cells were lysed for 20 min at room temperature and luciferase levels were measured with the Dual-Luciferase® Reporter 1000 assay system (Promega, ref. number E1980), according to the manufacturer’s protocol. Luminescence intensity was measured with a VICTOR-X Multilabel Plate Reader (PerkinElmer Life Sciences, USA). *Firefly* luciferase activity values were normalized against *Renilla* luciferase activity values. These values were then normalized against the median induction level for non-neutralizing samples, and expressed as a percentage. Samples were considered neutralizing if luciferase induction, normalized against *Renilla* luciferase activity, was below 15% of the median values for controls tested the same day.

### SARS-CoV-2 serological studies

#### Serum collection

Control serum was collected under informed consent from healthy recipients of BNT162b2 vaccine (vaccines based on the Wuhan spike protein -S protein- sequence), which were confirmed to have no prior SARS-CoV-2 infection by anti-SARS-CoV-2 nucleocapsid (N protein) IgG assay (*45*). All serum samples were heat inactivated at 56°C for 30 min prior to neutralization experiments.

#### Luminex Assay

Luminex immunoassays for SARS-CoV-2 serology studies were performed as previously described using proteins from the Wuhan strain of the virus (*46*). Briefly, whole N protein, trimeric Spike ectodomain (residues 1-1213) and receptor binding domain (residues 328-533, all generously provided by Dr. John Pak, Chan Zuckerberg Biohub) were each conjugated to a unique spectrally encoded bead using manufacturer instructions (Luminex Antibody Coupling Kit, #40-50016) with 5 μg of protein per 1 million beads. All beads were blocked overnight before use in PBST supplemented with 0.1% BSA and pooled on day of use. 2000-2500 beads per ID were pooled per replicate. Patient serum or plasma was incubated with beads at a final dilution of 1:250 for 1 hour, washed twice in PBST, stained with an anti-IgG (human) pre-conjugated to phycoerythrin (Thermo Scientific, #12-4998-82) for 30 min at 1:2000, then washed thrice in PBST. Primary incubations were done in PBST supplemented with 2% nonfat milk and secondary incubations were done in PBST. Beads were processed in duplicate in 96 well format and analyzed on a Luminex LX 200 cytometer. Median Fluorescence Intensity from each set of beads within each bead ID were retrieved directly from the LX200 after normalizing to the intra-assay negative controls (Bovine Serum Albumin (BSA) conjugated beads).

#### Pseudovirus production

SARS-CoV-2 pseudoviruses were generated using a previously described recombinant vesicular stomatitis virus expressing GFP in place of the VSV glycoprotein (rVSV∆G-GFP) (*47*). The SARS-CoV-2 spike gene bearing the D614G mutation or the set of mutations in the B.1.617.2/Delta variant (T19R, T95I, G142D, ∆157-158, L452R, T478K, P681R, D614G, D950N) were cloned in a CMV-driven expression vector and used to produce SARS-CoV-2 spike reporter pseudoviruses. Pseudoviruses were titered on Huh7.5.1 cells overexpressing ACE2 and Transmembrane protease, serine 2 (TMPRSS2) (gift of Andreas Puschnik) using GFP expression to measure the concentration of focus forming units (ffu).

#### Pseudovirus neutralization experiments

Huh7.5.1-ACE2-TMPRSS2 cells were seeded in 96-well plates at a density of 7000 cells/well one day prior to pseudovirus inoculation. Cells were verified to be free of mycoplasma contamination with the MycoAlert Mycoplasma detection kit (Lonza). Serum samples were diluted into complete culture media (DMEM with 10% FBS, 10mM HEPES, 1x Pen-Strep-Glutamine) using the LabCyte Echo 525 liquid handler and 1500 ffu of SARS-CoV-2 pseudovirus was added to each well to reach final dilutions ranging from 1:20-1:10240, including no-serum and no-pseudovirus controls. Serum/pseudovirus mixtures were incubated at 37°C for 1h before being added directly to cells. Cells inoculated with serum/pseudovirus mixtures were incubated at 37°C and 5% CO_2_ for 24h, resuspended using 10x TrypLE Select (Gibco), and cell fluorescence was measured with the BD Celesta flow cytometer. All neutralization assays were repeated for a total of three independent experiments with each experiment containing two technical replicates for each condition. Flow cytometry data was analyzed with FlowJo to determine the percentage of cells transduced with pseudovirus (GFP-positive). Percent neutralization for each serum dilution was calculated by normalizing GFP-positive cell percentage to no-serum control wells. Fifty percent neutralization titers (NT_50_) were calculated from ten-point response curves generated in GraphPad Prism 7 using four-parameter logistic regression.

## Appendix: List of COVID HGE Consortium authors

Laurent Abel^1^ Cristian Achille^2^ Alessandro Aiuti^3^ Saleh Al-Muhsen^4^ Fahd Al-Mulla^5^ Mark S. Anderson^6^ Evangelos Andreakos^7^ Micol Angelini^8^ Andrés A. Arias^9^ Gokhan Aytekin^10^ Fausto Baldanti^11^ Hagit Baris Feldman^12^ Alexandre Belot^13^ Federica Bergami^14^ Catherine M. Biggs^15^ Dusan Bogunovic^16^ Alexandre Bolze^17^ Anastasiia Bondarenko^18^ Ahmed A. Bousfiha^19^ Petter Brodin^20^ Yenan Bryceson^21^ Carlos D. Bustamante^22^ Manish J. Butte^23^ Giorgio Casari^24^ John Christodoulou^25^ Antonio Condino-Neto^26^ Stefan N. Constantinescu^27^ Francesca Conti^28^ Megan A. Cooper^29^ Clifton L. Dalgard^30^ Murkesh Desai^31^ Beth A. Drolet^32^ Jamila El Baghdadi^33^ Recai Ergun^34^ Dilek Ergun^35^ Sara Espinosa-Padilla^36^ Jacques Fellay^37^ Carlos Flores^38^ José Luis Franco^39^ Antoine Froidure^40^ Stefano Ghirardello^41^ Peter K. Gregersen^42^ Bodo Grimbacher^43^ Filomeen Haerynck^44^ David Hagin^45^ Rabih Halwani​^46^ Lennart Hammarström^47^ James R. Heath^48^ Sarah E. Henrickson^49^ Elena W.Y. Hsieh^50^ Eystein Husebye^51^ Kohsuke Imai^52^ Yuval Itan^53^ Erich D. Jarvis^54^ Fikret Kanat^55^ Timokratis Karamitros^56^ Kai Kisand^57^ Vasyl Kopcha^58^ Mykhaylo Korda^59^ Cheng-Lung Ku^60^ Yu-Lung Lau^61^ Yun Ling^62^ Carrie L. Lucas^63^ Tom Maniatis^64^ Davood Mansouri^65^ László Maródi^66^ Isabelle Meyts^67^ Joshua D. Milner^68^ Kristina Mironska^69^ Trine H. Mogensen^70^ Francesco Mojoli^71^ Francisco Morandeira^72^ Tomohiro Morio^73^ Lisa F.P. Ng^74^ Luigi D. Notarangelo^75^ Antonio Novelli^76^ Giuseppe Novelli^77^ Cliona O'Farrelly^78^ Satoshi Okada^79^ Keisuke Okamoto^80^ Tayfun Ozcelik^81^ Michele Pagani^82^ Qiang Pan-Hammarström^83^ Jean W. Pape^84^ Rebeca Perez de Diego^85^ David S. Perlin^86^ Graziano Pesole^87^ Andrea Pession^88^ Antonio Piralla^89^ Anna M. Planas^90^ Carolina Prando^91^ Aurora Pujol^92^ Lluis Quintana-Murci^93^ Sathishkumar Ramaswamy^94^ Laurent Renia^95^ Igor Resnick^96^ Raúl Rigo-Bonnin^97^ Carlos Rodríguez-Gallego^98^ Vanessa Sancho-Shimizu^99^ Anna Sediva^100^ Mikko R.J. Seppänen^101^ Mohammed Shahrooei^102^ Anna Shcherbina^103^ Ondrej Slaby^104^ Andrew L. Snow^105^ Pere Soler-Palacín^106^ András N. Spaan^107^ Ivan Tancevski^108^ Stuart G. Tangye^109^ Ahmad Abou Tayoun^110^ Baykal Tulek^111^ Stuart E. Turvey^112^ K M Furkan Uddin^113^ Mohammed Uddin^114^ Bénédicte Clément^115^

^1^Laboratory of Human Genetics of Infectious Diseases, Necker Branch, INSERM U1163, Necker Hospital for Sick Children, Paris, France; University of Paris, Imagine Institute, Paris, France. ^2^Neonatal Intensive Care Unit, Fondazione IRCCS Policlinico San Matteo, Pavia, Italy ^3^San Raffaele Telethon Institute for Gene Therapy, IRCCS Ospedale San Raffaele, and Vita Salute San Raffaele University, Milan, Italy. ^4^Immunology Research Lab, Department of Pediatrics, College of Medicine, King Saud University, Riyadh, Saudi Arabia. ^5^Dasman Diabetes Institute, Department of Genetics and Bioinformatics, Dasman, Kuwait. ^6^Diabetes Center, University of California San Francisco, San Francisco, CA, USA. ^7^Laboratory of Immunobiology, Center for Clinical, Experimental Surgery and Translational Research, Biomedical Research Foundation of the Academy of Athens, Athens, Greece. ^8^Neonatal Intensive Care Unit, Fondazione IRCCS Policlinico San Matteo, Pavia, Italy ^9^St. Giles Laboratory of Human Genetics of Infectious Diseases, Rockefeller Branch, The Rockefeller University, New York, NY, USA; Primary Immunodeficiencies Group, Department of Microbiology and Parasitology, School of Medicine, University of Antioquia, Medellín, Colombia; School of Microbiology, University of Antioquia UdeA, Medellín, Colombia. ^10^Department of Immunology and Allergy, Konya City Hospital ^11^Department of Clinical, Surgical, Diagnostic and Pediatric Sciences, University of Pavia, Pavia, Italy ^12^The Genetics Institute, Tel Aviv Sourasky Medical Center and Sackler Faculty of Medicine, Tel Aviv University, Tel Aviv, Israel. ^13^Pediatric Nephrology, Rheumatology, Dermatology, HFME, Hospices Civils de Lyon, National Referee Centre RAISE, and INSERM U1111, Université de Lyon, Lyon, France. ^14^Molecular Virology Unit, Microbiology and Virology Department, Fondazione IRCCS Policlinico San Matteo, Pavia, Italy ^15^Department of Pediatrics, BC Children's and St. Paul's Hospitals, University of British Columbia, Vancouver, BC, Canada. ^16^Icahn School of Medicine at Mount Sinai, New York, NY, USA. ^17^Helix, San Mateo, CA, USA. ^18^Shupyk National Healthcare University of Ukraine, Kyiv, Ukraine. ^19^Department of Pediatric Infectious Diseases and Clinical Immunology, CHU Ibn Rushd and LICIA, Laboratoire d'Immunologie Clinique, Inflammation et Allergie, Faculty of Medicine and Pharmacy, Hassan II University, Casablanca, Morocco. ^20^SciLifeLab, Department Of Women’s and Children’s Health, Karolinska Institutet, Stockholm, Sweden. ^21^Department of Medicine, Center for Hematology and Regenerative Medicine, Karolinska Institutet, Stockholm, Sweden. ^22^Stanford University, Stanford, CA, USA. ^23^Division of Immunology, Allergy, and Rheumatology, Department of Pediatrics and the Department of Microbiology, Immunology, and Molecular Genetics, University of California, Los Angeles, CA, USA. ^24^Clinical Genomics, IRCCS San Raffaele Scientific Institute and Vita-Salute San Raffaele University, Milan, Italy. ^25^Murdoch Children's Research Institute and Department of Pediatrics, University of Melbourne, Melbourne, VIC, Australia. ^26^Department of Immunology, Institute of Biomedical Sciences, University of São Paulo, São Paulo, Brazil. ^27^de Duve Institute and Ludwig Cancer Research, Brussels, Belgium. ^28^Pediatric Unit, IRCCS Azienda Ospedaliero-Universitaria di Bologna, Bologna, Italy ^29^Washington University School of Medicine, St. Louis, MO, USA. ^30^Department of Anatomy, Physiology & Genetics, Uniformed Services University of the Health Sciences, Bethesda, MD, USA. ^31^Bai Jerbai Wadia Hospital for Children, Mumbai, India. ^32^School of Medicine and Public Health, University of Wisconsin, Madison, WI, USA. ^33^Genetics Unit, Military Hospital Mohamed V, Rabat, Morocco. ^34^Selcuk University, Department of Pulmonology, Konya, Turkey ^35^Selcuk University, Department of Pulmonology, Konya, Turkey ^36^Instituto Nacional de Pediatria (National Institute of Pediatrics), Mexico City, Mexico. ^37^School of Life Sciences, Ecole Polytechnique Fédérale de Lausanne, Lausanne, Switzerland; Precision Medicine Unit, Lausanne University Hospital and University of Lausanne, Lausanne, Switzerland. ^38^Research Unit, Hospital Universitario Nuestra Señora de Candelaria, Santa Cruz de Tenerife; CIBER de Enfermedades Respiratorias, Instituto de Salud Carlos III, Madrid; Genomics Division, Instituto Tecnológico y de Energías Renovables (ITER), Santa Cruz de Tenerife, Spain. ^39^Group of Primary Immunodeficiencies, University of Antioquia UDEA, Medellin, Colombia. ^40^Pulmonology Department, Cliniques Universitaires Saint-Luc ; Institut de Recherche Expérimentale et Clinique (IREC), Université Catholique de Louvain, Brussels, Belgium. ^41^Neonatal Intensive Care Unit, Fondazione IRCCS Policlinico San Matteo, Pavia, Italy ^42^Feinstein Institute for Medical Research, Northwell Health USA, Manhasset, NY, USA. ^43^Center for Chronic Immunodeficiency & Institute for Immunodeficiency, Medical Center, Faculty of Medicine, University of Freiburg, Freiburg, Germany. ^44^Department of Pediatric Immunology and Pulmonology, Centre for Primary Immunodeficiency Ghent (CPIG), PID Research Laboratory, Jeffrey Model Diagnosis and Research Centre, Ghent University Hospital, Ghent, Belgium. ^45^The Genetics Institute Tel Aviv Sourasky Medical Center, Tel Aviv, Israel. ^46^Sharjah Institute of Medical Research, College of Medicine, University of Sharjah, Sharjah, United Arab Emirates. ^47^Department of Biosciences and Nutrition, Karolinska Institutet, Stockholm, Sweden. ^48^Institute for Systems Biology, Seattle, WA, USA. ^49^Department of Pediatrics, Division of Allergy Immunology, Children’s Hospital of Philadelphia, Philadelphia, PA, USA; Department of Microbiology, Perelman School of Medicine, University of Pennsylvania, Philadelphia, PA, USA. ^50^Departments of Pediatrics, Immunology and Microbiology, University of Colorado, School of Medicine, Aurora, CO, USA. ^51^Department of Medicine, Haukeland University Hospital, Bergen, Norway. ^52^Department of Community Pediatrics, Perinatal and Maternal Medicine, Tokyo Medical and Dental University (TMDU), Tokyo, Japan. ^53^Institute for Personalized Medicine, Icahn School of Medicine at Mount Sinai, New York, NY, USA; Department of Genetics and Genomic Sciences, Icahn School of Medicine at Mount Sinai, New York, NY, USA. ^54^Laboratory of Neurogenetics of Language and Howard Hughes Medical Institute, The Rockefeller University, New York, NY, USA. ^55^Selcuk University, Department of Pulmonology, Konya, Turkey ^56^Bioinformatics and Applied Genomics Unit, Hellenic Pasteur Institute, Athens, Greece. ^57^Molecular Pathology, Department of Biomedicine, Institute of Biomedicine and Translational Medicine, University of Tartu, Tartu Estonia. ^58^Horbachevsky Ternopil National Medical University ^59^Horbachevsky Ternopil National Medical University ^60^Chang Gung University, Taoyuan County, Taiwan. ^61^Department of Pediatrics & Adolescent Medicine, The University of Hong Kong, Hong Kong, China. ^62^Shanghai Public Health Clinical Center, Fudan University, Shanghai, China. ^63^Department of Immunobiology, Yale University School of Medicine, New Haven, CT, USA. ^64^Zukerman Mind Brain Behavior Institute, Columbia University, New York, NY, USA. ^65^Department of Clinical Immunology and Infectious Diseases, National Research Institute of Tuberculosis and Lung Diseases, The Clinical Tuberculosis and Epidemiology Research Center, National Research Institute of Tuberculosis and Lung Diseases (NRITLD), Masih Daneshvari Hospital, Shahid Beheshti, University of Medical Sciences, Tehran, Iran. ^66^Primary Immunodeficiency Clinical Unit and Laboratory, Department of Dermatology, Venereology and Dermatooncology, Semmelweis University, Budapest, Hungary. ^67^Department of Pediatrics, University Hospitals Leuven; KU Leuven, Department of Microbiology, Immunology and Transplantation; Laboratory for Inborn Errors of Immunity, KU Leuven, Leuven, Belgium. ^68^Department of Pediatrics, Columbia University Irving Medical Center, New York, NY, USA. ^69^University Clinic for Children's Diseases, Department of Pediatric Immunology, Medical Faculty, University “ St.Cyril and Methodij” Skopje, North Macedonia. ^70^Department of Biomedicine, Aarhus University, Aarhus, Denmark. ^71^Anesthesia and Intensive Care, Rianimazione I, Fondazione IRCCS Policlinico San Matteo, Pavia, Italy ^72^Department of Internal Medicine, Hospital Universitari de Bellvitge, IDIBELL, Barcelona, Spain. ^73^Tokyo Medical & Dental University Hospital, Tokyo, Japan. ^74^A*STAR Infectious Disease Labs, Agency for Science, Technology and Research, Singapore; Lee Kong Chian School of Medicine, Nanyang Technology University, Singapore. ^75^National Institute of Allergy and Infectious Diseases, National Institutes of Health, Bethesda, MD, USA. ^76^Laboratory of Medical Genetics, IRCCS Bambino Gesù Children’s Hospital, Rome, Italy. ^77^Department of Biomedicine and Prevention, Tor Vergata University of Rome, Rome, Italy. ^78^Comparative Immunology Group, School of Biochemistry and Immunology, Trinity Biomedical Sciences Institute, Trinity College Dublin, Ireland. ^79^Department of Pediatrics, Graduate School of Biomedical and Health Sciences, Hiroshima University, Hiroshima, Japan. ^80^Tokyo Medical and Dental University, Tokyo, Japan. ^81^Department of Molecular Biology and Genetics, Bilkent University, Bilkent - Ankara, Turkey. ^82^Anesthesia and Intensive Care, Rianimazione I, Fondazione IRCCS Policlinico San Matteo, Pavia, Italy ^83^Department of Biosciences and Nutrition, Karolinska Institutet, Stockholm, Sweden. ^84^Haitian Study Group for Kaposi's Sarcoma and Opportunistic Infections (GHESKIO), Port-au-Prince, Haiti. ^85^Institute of Biomedical Research of IdiPAZ, University Hospital “La Paz”, Madrid, Spain. ^86^Center for Discovery and Innovation, Hackensack Meridian Health, Nutley, NJ, USA. ^87^Department of Biosciences, Biotechnology and Biopharmaceutics, University of Bari A. Moro, Bari, Italy. ^88^Pediatric Unit, IRCCS Azienda Ospedaliero-Universitaria di Bologna, Bologna, Italy ^89^Molecular Virology Unit, Microbiology and Virology Department, Fondazione IRCCS Policlinico San Matteo, Pavia, Italy ^90^IIBB-CSIC, IDIBAPS, Barcelona, Spain. ^91^Faculdades Pequeno Príncipe, Instituto de Pesquisa Pelé Pequeno Príncipe, Curitiba, Brazil. ^92^Neurometabolic Diseases Laboratory, Bellvitge Biomedical Research Institute (IDIBELL), L'Hospitalet de Llobregat, Barcelona, Spain; Catalan Institution of Research and Advanced Studies (ICREA), Barcelona, Spain; Center for Biomedical Research on Rare Diseases (CIBERER), ISCIII, Barcelona, Spain. ^93^Human Evolutionary Genetics Unit, CNRS U2000, Institut Pasteur, Paris, France; Human Genomics and Evolution, Collège de France, Paris, France. ^94^Al Jalila Children's Hospital, Dubai, UAE. ^95^A*STAR Infectious Disease Labs, Agency for Science, Technology and Research, Singapore; Lee Kong Chian School of Medicine, Nanyang Technology University, Singapore. ^96^University Hospital St. Marina, Varna, Bulgaria. ^97^Department of Clinical Laboratory, Hospital Universitari de Bellvitge, IDIBELL, Barcelona, Spain. ^98^Department of Immunology, University Hospital of Gran Canaria Dr. Negrín, Canarian Health System, Las Palmas de Gran Canaria; Department of Clinical Sciences, University Fernando Pessoa Canarias, Las Palmas de Gran Canaria, Spain. ^99^Department of Pediatric Infectious Diseases and Virology, Imperial College London, London, UK; Centre for Pediatrics and Child Health, Faculty of Medicine, Imperial College London, London, UK. ^100^Department of Immunology, Second Faculty of Medicine Charles University, V Uvalu, University Hospital in Motol, Prague, Czech Republic. ^101^Adult Immunodeficiency Unit, Infectious Diseases, Inflammation Center, University of Helsinki and Helsinki University Hospital, Helsinki, Finland; Rare Diseases Center and Pediatric Research Center, Children's Hospital, University of Helsinki and Helsinki University Hospital, Helsinki, Finland. ^102^Specialized Immunology Laboratory of Dr. Shahrooei, Ahvaz, Iran; Department of Microbiology and Immunology, Clinical and Diagnostic Immunology, KU Leuven, Leuven, Belgium. ^103^Department of Immunology, Dmitry Rogachev National Medical Research Center of Pediatric Hematology, Oncology and Immunology, Moscow, Russia. ^104^Central European Institute of Technology & Department of Biology, Faculty of Medicine, Masaryk University, Brno, Czech Republic. ^105^Department of Pharmacology & Molecular Therapeutics, Uniformed Services University of the Health Sciences, Bethesda, MD, USA. ^106^Pediatric Infectious Diseases and Immunodeficiencies Unit, Vall d’Hebron Barcelona Hospital Campus, Barcelona, Catalonia, Spain. ^107^St. Giles Laboratory of Human Genetics of Infectious Diseases, Rockefeller Branch, The Rockefeller University, New York, NY, USA; Department of Medical Microbiology, University Medical Center Utrecht, Utrecht, Netherlands. ^108^Department of Internal Medicine II, Medical University of Innsbruck, Innsbruck, Austria. ^109^Garvan Institute of Medical Research, Darlinghurst, NSW, Australia; St Vincent’s Clinical School, Faculty of Medicine, UNSW Sydney, NSW, Australia. ^110^Al Jalila Children's Hospital, Dubai, UAE. ^111^Selcuk University, Department of Pulmonology, Konya, Turkey ^112^BC Children's Hospital, The University of British Columbia, Vancouver, Canada. ^113^Centre for Precision Therapeutics, Genetics & Genomic Medicine Centre, NeuroGen Children's Healthcare and Lecturer, Holy Family Red Crescent Medical College Dhaka, Bangladesh. ^114^ Mohammed Bin Rashid University of Medicine and Health Sciences, Dubai, UAE; GenomeArc Inc., Toronto, ON, Canada. ^115^ Service des Urgences, Groupement Hospitalier Nord, Hospices Civils de Lyon, Lyon, France.

## Appendix: List of COMET Consortium authors

Yumiko Abe-Jones, MS^1^, Saurabh Asthana, PhD^2,3,4^, Sharvari Bhide, BA^5^, Carolyn S. Calfee, M ^6,7^, Sidney A. Carrillo, MPH^6^, Suzanna Chak, BA^6^, Zachary Collins, BA^2,3,4^, David J. Erle, MD^3,4,7,8^, Gabriela K. Fragiadakis, PhD^3,4,9^, Rajani Ghale, MS^6^, Carolyn M. Hendrickson, MD^5,8^, Alejandra Jauregui, BA^6^, Kirsten N. Kangelaris, MD^1^, Matthew F. Krummel, PhD^2,3,4^, Charles R. Langelier, MD, PhD^10,11^, Tasha Lea, MS^2^, Deanna Lee, BA^5,8^, Aleksandra Leligdowicz, MD, PhD^12^, Carolyn Leroux, BS^6^, Raphael Lota, BA^13^, Michael Matthay, MD^7^, Viet Nguyen, BA^5,8^, Ravi Patel, PhD^3,4^, Logan Pierce, MD^1^, Priya Prasad, PhD^1^, Arjun Arkal Rao, PhD^2,3,4^, Ahmad Rashid, BS^13^, Nicklaus Rodriguez, BA^13^, Bushra Samad, MS^2,3,4^, Cole Shaw, MSEE^2^ ^,^ ^3^ ^,^ ^4^, Austin Sigman, BS^6^, Kevin Tang, MS^13^, Luz Torres Altamirano, BA^13^, Alyssa Ward, PhD^9^, Andrew Willmore, BS^6^, Michael Wilson, MD^14^, Prescott G. Woodruff, MD^6,7^

1. Division of Hospital Medicine, University of California, San Francisco, CA, USA.

2. Department of Pathology, University of California, San Francisco, CA, USA.

3. UCSF CoLabs, University of California, San Francisco, CA, USA.

4. Bakar ImmunoX Initiative, University of California, San Francisco, CA, USA.

5. Division of Pulmonary and Critical Care Medicine, Department of Medicine, Zuckerberg San Francisco General Hospital and Trauma Center, University of California, San Francisco, CA, USA.

6. Division of Pulmonary and Critical Care Medicine, Department of Medicine, University of California San Francisco, San Francisco, California, USA.

7. Cardiovascular Research Institute, University of California, San Francisco, San Francisco, CA, USA.

8. Lung Biology Center, University of California, San Francisco, San Francisco, CA, USA

9. Division of Rheumatology, Department of Medicine, University of California, San Francisco, CA, USA.

10. Division of Infectious Diseases, University of California, San Francisco, CA, USA.

11. Chan Zuckerberg Biohub, University of California, San Francisco, CA, USA.

12. Division of Critical Care Medicine, Robarts Research Institute, University of Western Ontario, London, ON, Canada.

13. Helen Diller Family Comprehensive Cancer Center, University of California, San Francisco, CA, USA.

14. Weill Institute for Neurosciences, Department of Neurology, University of California, San Francisco, CA, USA.

## Appendix: List of French COVID study group authors

Laurent ABEL^1^, Clotilde ALLAVENA^2^, Claire ANDREJAK^3^, François ANGOULVANT^4^, Cecile AZOULAY^5^, Delphine BACHELET^6^, Marie BARTOLI^7^, Romain BASMACI^8^, Sylvie BEHILILL^9^, Marine BELUZE^10^, Nicolas BENECH^11^, Dehbia BENKERROU^12^, Krishna BHAVSAR^6^, Laurent BITKER^11^, Lila BOUADMA^6^, Maude BOUSCAMBERT^13^, Pauline CARAUX PAZ^14^, Minerva CERVANTES-GONZALEZ^6^, Anissa CHAIR^6^, Catherine CHIROUZE^15^, Alexandra COELHO^16^, Hugues CORDEL^17^, Camille COUFFIGNAL^6^, Sandrine COUFFIN-CADIERGUES^18^, Eric d’ORTENZIO^7^, Etienne DE MONTMOLLIN^6^, Alexa DEBARD^19^, Marie-Pierre DEBRAY^6^, Dominique DEPLANQUE^20^, Diane DESCAMPS^6^, Mathilde DESVALLÉE^21^, Alpha DIALLO^7^, Jean-Luc DIEHL^22^, Alphonsine DIOUF^16^, Céline DORIVAL^12^, François DUBOS^23^, Xavier DUVAL^6^, Philippine ELOY^6^, Vincent ENOUF^9^, Olivier EPAULARD^24^, Hélène ESPEROU^18^, Marina ESPOSITO-FARESE^6^, Manuel ETIENNE^25^, Denis GAROT^26^, Nathalie GAULT^6^, Alexandre GAYMARD^13^, Jade GHOSN^6^, Tristan GIGANTE^27^, Morgane GILG^27^, François GOEHRINGER^28^, Jérémie GUEDJ^29^, Alexandre HOCTIN^16^, Isabelle HOFFMANN^6^, Ikram HOUAS^18^, Jean-Sébastien HULOT^22^, Salma JAAFOURA^18^, Ouifiya KAFIF^6^, Florentia KAGUELIDOU^30^, Sabrina KALI^6^, Younes KERROUMI^31^, Antoine KHALIL^6^, Coralie KHAN^21^, Antoine KIMMOUN^28^, Fabrice LAINE^32^, Cédric LAOUÉNAN^6^, Samira LARIBI^6^, Minh LE^6^, Cyril LE BRIS^33^, Sylvie LE GAC ^6^, Quentin LE HINGRAT^6^, Soizic LE MESTRE^7^, Hervé LE NAGARD^29^, Adrien LEMAIGNEN^26^, Véronique LEMEE^25^, François-Xavier LESCURE^6^, Sophie LETROU^6^, Yves LEVY^34^, Bruno LINA^13^, Guillaume LINGAS^29^, Jean Christophe LUCET^6^, Moïse MACHADO^35^, Denis MALVY^36^, Marina MAMBERT^16^, Aldric MANUEL^37^, France MENTRÉ^6^, Amina MEZIANE^12^, Hugo MOUQUET^9^, Jimmy Mullaert^6^, Nadège NEANT^29^, Duc NGUYEN^36^, Marion NORET^38^, Aurélie PAPADOPOULOS^18^, Christelle PAUL^7^, Nathan PEIFFER-SMADJA^6^, Vincent PEIGNE^39^, Ventzislava PETROV-SANCHEZ^7^, Gilles PEYTAVIN^6^, Huong PHAM^6^, Olivier PICONE^8^, Valentine PIQUARD^6^, Julien POISSY^23^, Oriane PUÉCHAL^40^, Manuel ROSA-CALATRAVA^13^, Bénédicte ROSSIGNOL^27^, Patrick ROSSIGNOL^28^, Carine ROY^6^, Marion SCHNEIDER^6^, Richa SU^6^, Coralie TARDIVON^6^, Marie-Capucine TELLIER^6^, François TÉOULÉ^12^, Olivier TERRIER^13^, Jean-François TIMSIT^6^, Christelle TUAL^41^, Sarah TUBIANA^6^, Sylvie VAN DER WERF^9^, Noémie VANEL^42^, Aurélie VEISLINGER^41^, Benoit VISSEAUX^6, 29^, Aurélie WIEDEMANN^34^, Yazdan YAZDANPANAH^6^

^1^Inserm UMR 1163, Paris, France. ^2^CHU Nantes, France. ^3^CHU Amiens, France. ^4^Hôpital Necker, Paris, France. ^5^Hopitâl Cochin, Paris, France. ^6^Hôpital Bichat, Paris, France. ^7^ANRS, Paris, France. ^8^Hôpital Louis Mourier, Colombes, France. ^9^Pasteur Institute, Paris, France. ^10^F-CRIN Partners Platform, Paris, France. ^11^CHU Lyon, France. ^12^Inserm UMR 1136, Paris, France. ^13^Inserm UMR 1111, Lyon, France. ^14^CH Villeneuve Saint Georges, France. ^15^CHRU Jean Minjoz, Besançon, France. ^16^Inserm UMR 1018, Paris, France. ^17^Hôpital Avicenne, Bobigny, France. ^18^Inserm sponsor, Paris, France. ^19^CHU Toulouse, France. ^20^CIC 1403 Inserm - CHU Lille, France. ^21^Inserm UMR 1219, Bordeaux, France. ^22^Hôpital Européen Georges Pompidou, Paris, France. ^23^CHU Lille, France. ^24^CHU Grenoble, France. ^25^CHU Rouen, France. ^26^CHU Tours, France. ^27^F-CRIN INI-CRCT, Nancy, France. ^28^CHU Nancy, France. ^29^Inserm UMR 1137, Paris, France. ^30^Hôpital Robert Debré, Paris, France. ^31^GH Diaconesses, Paris, France. ^32^CHU Rennes, France. ^33^CH Beziers, France. ^34^Vaccine Research Insitute (VRI), Inserm UMR ^955^, Créteil, France. ^35^Grand Hôpital de l’Est Francilien, Marne-la-Vallée, France. ^36^CHU Bordeaux, France. ^37^CH Annecy, France. ^38^RENARCI, Annecy, France. ^39^CH Métropole Savoie, Cambery, France. ^40^REACTing, Paris, France. ^41^Inserm CIC-1414, Rennes, France. ^42^Hôpital la Timone, Marseille, France.
