# Supplementary material for: Vaccine breakthrough hypoxemic COVID-19 pneumonia in patients with auto-Abs neutralizing type I IFNs
Source: Sci Immunol. 2022 Jun 14:eabp8966. doi: 10.1126/sciimmunol.abp8966 (PMC9210448; doi:10.1126/sciimmunol.abp8966)
Supplement: Supplementary file 1 — Materials and Methods Fig. S1 Table S1 [file sciimmunol.abp8966_sm.pdf]

Science Immunology
AAAS

Supplementary Materials for

Vaccine breakthrough hypoxemic COVID-19 pneumonia in patients with auto-Abs neutralizing type I IFNs

Paul Bastard *et al.*

Corresponding authors: Mark S. Anderson, Mark.Anderson@ucsf.edu; Jean-Laurent Casanova, jean-laurent.casanova@rockefeller.edu; Joseph L. DeRisi, jderisi@gmail.com

DOI: 10.1126/sciimmunol.abp8966

The PDF file includes:

Materials and Methods
Fig. S1
Table S1

Other Supplementary Material for this manuscript includes the following:

Data file S1

Supplementary Materials for

Vaccine breakthrough hypoxemic COVID-19 pneumonia in patients with auto-Abs neutralizing type I IFNs

Paul Bastard[1-4,*], Sara Vazquez[5-7,*], Jamin Liu[8,9,*], Matthew T. Laurie[8,*], Chung Yu Wang[10,*], Adrian Gervais [1,2,*], Tom Le Voyer [1,2,*], Lucy Bizien[1,2,$], Colin Zamecnik[11,$], Quentin Philippot[1,2,$], Jérémie Rosain[1,2,$], Emilie Catherinot[12], Andrew Willmore[10], Anthea M. Mitchell[10], Rebecca Bair[11], Pierre Garçon[13], Heather Kenney[14], Arnaud Fekkar[1,2,15], Maria Salagianni[16], Garyphallia Poulakou[17], Eleni Siouti[16], Sabina Sahanic[18], Ivan Tancevski[18], Günter Weiss[18], Laurenz Nagl[19], Jérémy Manry[1,2], Sotirija Duvlis[20,21], Daniel Arroyo-Sánchez[22], Estela Paz Artal[22], Luis Rubio[8], Cristiano Perani[23], Michela Bezzi[24], Alessandra Sottini[25], Virginia Quaresima[25], Lucie Roussel[26,27], Donald C. Vinh[26,27], Luis Felipe Reyes[28,29], Margaux Garzaro[30], Nevin Hatipoglu[31], David Boutboul[32], Yacine Tandjaoui-Lambiotte[33-35], Alessandro Borghesi[36], Anna Aliberti[37], Irene Cassaniti[38], Fabienne Venet[39-41], Guillaume Monneret[39,40], Rabih Halwani[42,43], Narjes Saheb Sharif-Askari[42], Jeffrey Danielson[14], Sonia Burrel[44], Caroline Morbieu[45], Yurii Stepanovskyy[46], Anastasia Bondarenko[46], Alla Volokha[46], Oksana Boyarchuk[47], Alenka Gagro[48], Mathilde Neuville[49], Bénédicte Neven[50], Sevgi Keles[51], Romain Hernu[52], Antonin Bal[53], Antonio Novelli[54], Giuseppe Novelli[55], Kahina Saker[56], Oana Ailioaie[57], Arnau Antolí[58], Eric Jeziorski[59], Gemma Rocamora-Blanch[58], Carla Teixeira[60], Clarisse Delaunay[61], Marine Lhuillier[62], Paul Le Turnier[61], Yu Zhang[14,63], Matthieu Mahevas[64-66], Qiang Pan-Hammarström[67], Hassan Abolhassani[67], Thierry Bompoil, Ph.D, [68], Karim Dorgham[69], COVID HGE consortium[†], French COVID study group[†], COMET consortium[†], Guy Gorochov[69,70], Cédric Laouenan[33,71,72], Carlos Rodríguez-Gallego[,73,74], Lisa F. P. Ng[75], Laurent Renia[75-77], Aurora Pujol[78], Alexandre Belot[56,79], François Raffi[61], Luis M. Allende[22], Javier

Martinez-Picado[80-83], Tayfun Ozcelik[84], Sevgi Keles[51], Luisa Imberti[25], Luigi D. Notarangelo[14], Jesus Troya[85], Xavier Solanich[58], Shen-Ying Zhang[1-3], Anne Puel[1-3], Michael R Wilson[11], Sophie Trouillet-Assant[86], Laurent Abel[1-3], Emmanuelle Jouanguy[1-3], Chun Jimmie Ye[10,87-90,§], Aurélie Cobat[1-3,§], Leslie M. Thompson[91,§], Evangelos Andreakos[16,§], Qian Zhang[1-3,§], Mark S. Anderson[7,92,#,@], Jean-Laurent Casanova[1-4,93,#,@] and Joseph L. DeRisi[8,10,#,@]

[*]These authors contributed equally to this work.
[$]These authors contributed equally to this work.
[§]These authors contributed equally to this work.
[#]These authors contributed equally to this work.

[†]All collaborators and their affiliations appear at the end of this paper.

[@]corresponding authors: Mark.Anderson@ucsf.edu; jean-laurent.casanova@rockefeller.edu; jderisi@gmail.com

This PDF file includes:

Supplementary table S1 and legends to supplementary table (S1)
Legends to supplementary figure S1
Supplementary figure S1

Supplementary materials and methods

Subjects and samples

We enrolled, from 6 countries, 48 patients with proven hypoxemic COVID-19 pneumonia, 12 unvaccinated controls, and 11 vaccinated controls in this study. We collected plasma or serum samples for all these individuals to test by immuno-assay for the presence of IgG Abs against SARS-CoV-2 and auto-Abs to type I IFNs. All individuals were recruited according to protocols approved by local Institutional Review Boards (IRBs). For patients were enrolled in the French COVID cohort (clinicaltrials.gov NCT04262921), ethics approval was obtained from the CPP IDF VI (ID RCB: 2020-A00256-33). For patients enrolled at UCSF, IRB number was: 20-30497. All protocols conformed to local ethics recommendations and informed consent was obtained when required.

Table S1: Antibody response to the vaccine

	**Spike p-val**	**RBD p-val**
NOVAX vs POSTVAX	8,46E-18	8,98E-15
NOVAX vs all breakthrough (n=40)	1,95E-06	0,000147706
aab+ (n=10) vs aab- breakthrough (n=30)	0,4320157	0,259816689

Figure S1: No neutralizing auto-Abs against IFN-α2 and IFN-ω in patients with hypoxemic breakthrough COVID-19 and an insufficient serological response or immunodeficiencies. Serological response in auto-Ab positive patients. (A) Spike(S)-protein and receptor binding domain (RBD) serological titers, plotted against each other for breakthrough COVID-19 patients **(B)** Neutralization of 10 ng/mL IFN-α2, IFN-ω or IFN-β in the presence of plasma 1/10 from patients with hypoxemic breakthrough COVID-19 and previously known immunodeficiency (N=5) or with low Ab response to the virus (n=1). The patients with low Ab response to the virus is shown with an arrow. Relative luciferase activity is shown (ISRE dual luciferase activity, with normalization against *Renilla* luciferase activity) after stimulation with 10ng/mL IFN-α2 or IFN-ω in the presence of plasma 1/10. RLA: relative luciferase activity. **(C)** Neutralization of 100 pg/mL IFN-α2 or IFN-ω in the presence of plasma 1/10 from patients with hypoxemic breakthrough COVID-19 and previously known immunodeficiency (N=5) or with low Ab response to the virus (n=1). **(D)** Spike(S)-protein and receptor binding domain (RBD) serological titers of the 10 auto-Ab positive patients. **(E)** Spike(S)-protein and receptor binding domain (RBD) serological titers of the 10 auto-Ab positive patients, represented individually.

Figure S1

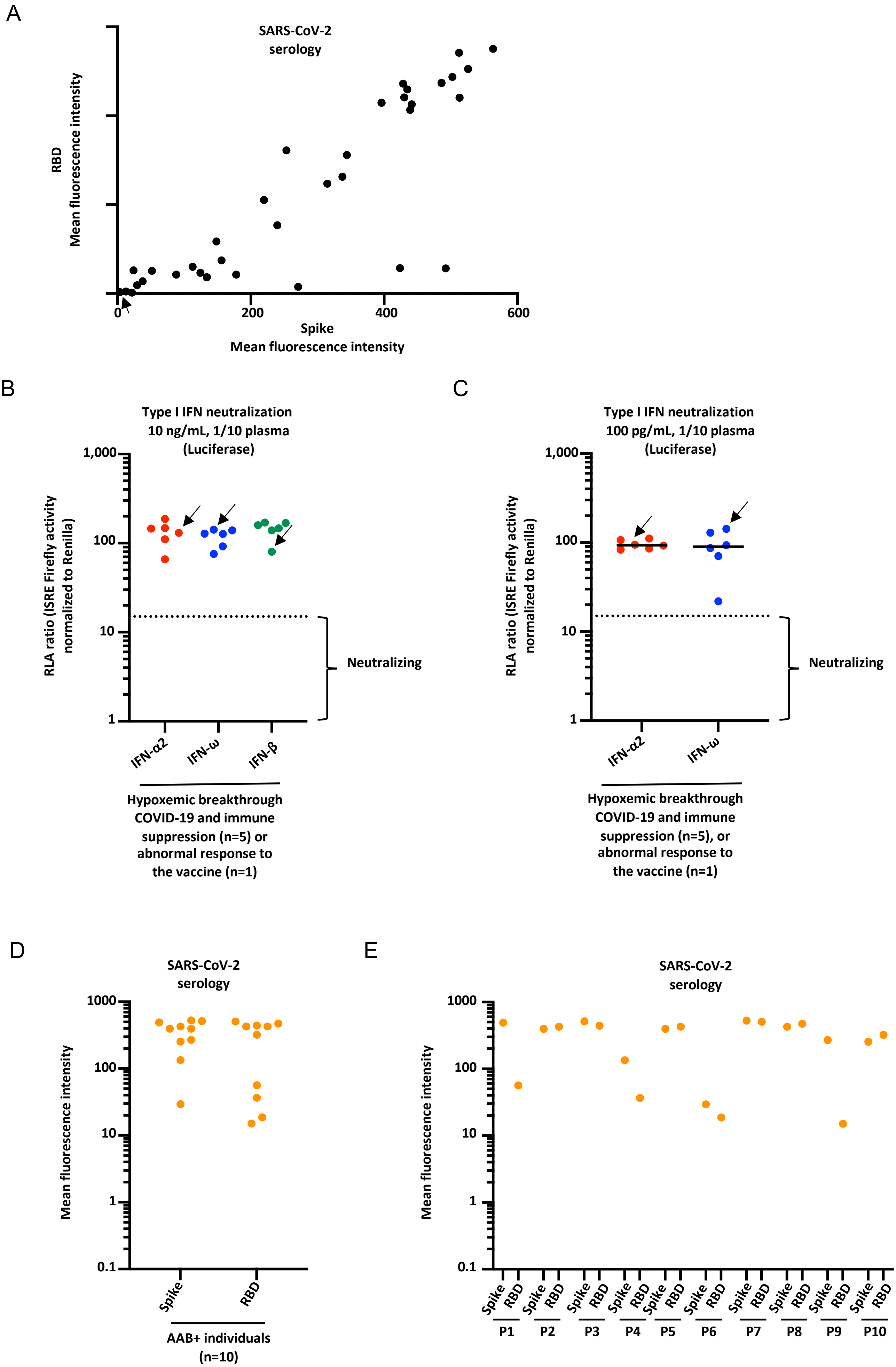

A
SARS-CoV-2
serology
RBD
Mean fluorescence intensity
0
200
400
600
Spike
Mean fluorescence intensity
B
Type I IFN neutralization
10 ng/mL, 1/10 plasma
(Luciferase)
1,000
100
10
1
RLA ratio (ISRE Firefly activity
normalized to Renilla)
Neutralizing
IFN-α2
IFN-ω
IFN-β
Hypoxemic breakthrough
COVID-19 and immune
suppression (n=5) or
abnormal response to
the vaccine (n=1)
C
Type I IFN neutralization
100 pg/mL, 1/10 plasma
(Luciferase)
1,000
100
10
1
RLA ratio (ISRE Firefly activity
normalized to Renilla)
Neutralizing
IFN-α2
IFN-ω
Hypoxemic breakthrough
COVID-19 and immune
suppression (n=5), or
abnormal response to
the vaccine (n=1)
D
SARS-CoV-2
serology
1000
100
10
1
0.1
Mean fluorescence intensity
Spike
RBD
AAB+ individuals
(n=10)
E
SARS-CoV-2
serology
1000
100
10
1
0.1
Mean fluorescence intensity
Spike
RBD
P1
P2
P3
P4
P5
P6
P7
P8
P9
P10
